# Editorial: Emerging talents in primary immunodeficiencies: 2022

**DOI:** 10.3389/fimmu.2023.1267778

**Published:** 2023-11-23

**Authors:** Yu Lung Lau, Andrew Gennery

**Affiliations:** ^1^ Department of Paediatrics & Adolescent Medicine, School of Clinical Medicine, LKS Faculty of Medicine, The University of Hong Kong, Hong Kong, Hong Kong SAR, China; ^2^ Translational and Clinical Research Institute, Newcastle University, Newcastle upon Tyne, United Kingdom; ^3^ Paediatric Stem Cell Transplant Unit, Great North Children’s Hospital, Newcastle upon Tyne, United Kingdom

**Keywords:** consanguinity, emapalumab, post-transplant cyclophosphamide, IEI registry, international collaboration

The Research Topic for this series of 6 articles is “*Emerging Talents in Primary Immunodeficiencies 2022*”. The goal of this Research Topic is to facilitate the young doctors and scientists to publish in the field of inborn errors of immunity (IEI), previously known as primary immunodeficiencies. The development of the care of patients with IEI differs enormously from country to country which is correlated with the Human Development Index (HDI) (1). The less resourced countries with low HDI need help from the more resourced countries with high HDI through networking with regional societies of immunodeficiencies such as European Society of Immunodeficiencies (ESID) and Asia Pacific Society for Immunodeficiencies (APSID) to provide training and collaborate so as to improve care of patients with IEI. Regardless of the geographical area from which they come, however, young researchers in the field face similar challenges – finding mentors, obtaining funding for projects, publishing and getting recognition in the field. Whilst those in HDI countries may have better access to funding, nevertheless, the first stages of this journey can be difficult.

Of the 6 articles, 2 are case reports from the less resourced countries with low HDI, Sudan and Kenya, which described their first case of autosomal recessive *IL12RB1* mutation and X-linked chronic granulomatous disease (XL-CGD), respectively. Abdelmajeed et al. from Sudan described a 4-generations pedigree with 6 first-degree consanguineous marriages, with many affected members highlighting the complex population structures and high consanguinity rate up to 67% in some parts of Sudan. Autosomal recessive IEI should be common and pre-marriage counseling could play a role in improving patient outcomes in such settings. Marangu-Boore et al. from Kenya described a boy with XL-CGD who was diagnosed and treated with haplo-identical hematopoietic stem cell transplantation (HSCT) using resources from a genetic laboratory in United States as well as clinical expertise including HSCT in South Africa and India. This case illustrated the potential of international collaboration in improving care for IEI patients in less resourced countries.

The remaining 4 articles are from countries with high HDI, i.e. Italy, Spain, United Kingdom and United States. Dell’Orso et al. from Italy reported their 30-year single-centre experience of HSCT for 67 patients with IEI. Improved outcomes through the years were observed and likely due to introduction of the TCR ab/CD19-depleted platform, hence reducing severe acute graft-versus-host disease as well as ensuring a donor is nearly always available with using a haploidentical family donor (HIFD). We need to find means to introduce effective HSCT for patients with IEI in less resourced countries in Asia and Africa. Instead of using TCR ab/CD-19 depleted platform which is expensive, post-transplant cyclophosphamide has been shown to be feasible to enable use of HIFD in haploidentical HSCT for patients with IEI (2).

Daza-Cajigal et al. from Spain reported the results of a survey of 38 patients with common variable immunodeficiency (CVID) related liver disease in 4 Spanish hospitals. Since Spain has a population of 47 million, there should be more patients with CVID-related liver disease, hence highlighting the importance to have national registries of patients with IEI to enable national studies of rare diseases such as IEI in order to have enough number of patients for impactful analysis. Again collaboration is the key for better research leading to improved patient care.

The final 2 articles review important topics in the field. Tsilifis et al. review the growing area of primary immune regulatory disorders, and focus on specific disorders, namely autoimmune polyendocrinopathy-candidiasis-ectodermal dystrophy, autoimmune lymphoproliferative syndromes, IPEX syndrome, CTLA-4 haploinsufficiency and LRBA and DEF6 deficiencies, IL-2RA and IL-2RB deficiencies, STAT3 gain-of-function, HLH syndromes, very early onset inflammatory bowel diseases, and activated phosphoinositide-3 kinase δ syndrome. Given the multi-system presentation, variable penetrance within families and gradual onset of manifestations, these diseases can be difficult to diagnose, but as targeted therapies become available, an awareness of the diseases and early diagnosis becomes important.

Lastly Steen et al. from United States wrote a review on the cellular pathophysiology of familial hemophagocytic lymphohistocytosis (fHLH) based on murine models of fHLH. This article highlighted that the understanding of disease mechanisms through collaboration with basic scientists studying animal models could shed lights on how to improve treatment outcome through targeted therapy. Targeting the dysregulated pathway of increased interferon- γ in fHLH, emapalumab, a fully humanised IgG1 anti-interferon-γ monoclonal antibody, has been shown to improve outcome of children with fHLH (3).

In conclusion these 6 authors have demonstrated their enthusiasm and dedication to improve their patient’s outcome through meticulous approach in clinical and scientific pursuit and their articles highlighted the importance of collaboration.

## Author contributions

YL: Writing – original draft, Conceptualization, Writing – review & editing. ARG: Writing – review & editing.
